# Long-term Efficacy, Safety, and Immunogenicity of the Infliximab (IFX) Biosimilar, PF-06438179/GP1111, in Patients with Rheumatoid Arthritis After Switching from Reference IFX or Continuing Biosimilar Therapy: Week 54–78 Data From a Randomized, Double-Blind, Phase III Trial

**DOI:** 10.1007/s40259-019-00403-z

**Published:** 2020-01-14

**Authors:** Stanley B. Cohen, Sebastiao C. Radominski, Hideto Kameda, Alan J. Kivitz, Michael Tee, Carol Cronenberger, Min Zhang, Sarah Hackley, Muhammad I. Rehman, Oliver von Richter, Rieke Alten

**Affiliations:** 1 Metroplex Clinical Research Center, Dallas, TX USA; 2 Universidade Federal do Paraná, Rua General Carneiro, 181-Alto Da Glória, Curitiba, PR 80060-900 Brazil; 3 Toho University, 2-22-36, Ohashi Meguro-ku, Tokyo, 153-8515 Japan; 4 Altoona Center for Clinical Research, Duncansville, PA USA; 5 Department of Medicine, Medical Center Manila, University of the Philippines, Manila, Philippines; 6 Pfizer Inc, Collegeville, PA USA; 7 Pfizer Inc, La Jolla, CA USA; 8 Pfizer R&D UK, Ltd, Sandwich, Kent, UK; 9 Pfizer Inc, Andover, MA USA; 10 Biopharmaceuticals, Hexal AG (a Sandoz company), Holzkirchen, Germany; 11 Schlosspark-Klinik University Medicine, Heubnerweg 2, 14059 Berlin, Germany

## Abstract

**Objective:**

Our objective was to evaluate the long-term efficacy, safety, and immunogenicity of the infliximab biosimilar, PF-06438179/GP1111 (PF-SZ-IFX), in patients with rheumatoid arthritis (RA) who continued biosimilar treatment throughout 78 weeks or who switched from reference infliximab (Remicade^®^) sourced from the EU (IFX-EU) at week 30 or week 54 in the REFLECTIONS B537-02 study.

**Methods:**

In this phase III, double-blind, active-controlled study, patients with moderate-to-severe active RA were initially randomized to PF-SZ-IFX or IFX-EU, each with methotrexate (treatment period [TP] 1; *N* = 650). At week 30, patients receiving PF-SZ-IFX continued PF-SZ-IFX; patients receiving IFX-EU were re-randomized to continue IFX-EU or switch to PF-SZ-IFX (TP2; *n* = 566). From weeks 54 to 78, all patients received open-label treatment with PF-SZ-IFX (TP3; *n* = 505). Efficacy, safety, and immunogenicity data were analyzed during TP3.

**Results:**

Efficacy was sustained and comparable across groups at week 78, with American College of Rheumatology criteria for ≥ 20% clinical improvement response rates of 75.9% (biosimilar group), 77.8% (week 30 switch group), and 68.3% (week 54 switch group). The incidence of treatment-emergent adverse events was 28.9%, 29.4%, and 30.2%, respectively. The proportion of patients who were antidrug antibody (ADA) positive and neutralizing antibody positive (as a percentage of ADA-positive patients) was stable and comparable between groups.

**Conclusions:**

Results to week 78 continue to support the efficacy, safety, and immunogenicity of PF-SZ-IFX in patients with moderate-to-severe active RA. There were no clinically meaningful differences between groups, independent of a single treatment transition from IFX-EU to PF-SZ-IFX at week 30 or week 54.

**Trial Registration Number:**

NCT02222493.

**Electronic supplementary material:**

The online version of this article (10.1007/s40259-019-00403-z) contains supplementary material, which is available to authorized users.

## Key Points

**Table:** 

Patients with moderate-to-severe active rheumatoid arthritis (RA) receiving PF-06438179/GP1111 (PF-SZ-IFX), an infliximab biosimilar, experienced no clinically meaningful differences in efficacy, safety, or immunogenicity, regardless of whether they were maintained on PF-SZ-IFX throughout 78 weeks of treatment, or following single treatment transitions from reference infliximab (Remicade^®^) sourced from the EU (IFX-EU) to PF-SZ-IFX at week 30 or at week 54.
PF-SZ-IFX was well-tolerated for up to 78 weeks of treatment and displayed a safety profile consistent with that of infliximab.
These findings provide long-term clinical data for PF-SZ-IFX to add to the “totality of the evidence” supporting the biosimilarity of PF-SZ-IFX to reference infliximab and its use in the other eligible indications for which reference infliximab is authorized.

## Introduction

The chimeric monoclonal antibody infliximab (Remicade^®^; Janssen Biotech, Horsham, PA, USA; Janssen Biologics B.V., Leiden, the Netherlands) is a tumor necrosis factor (TNF)-α inhibitor approved for the treatment of a range of immune-related inflammatory diseases [1–3]. In the two decades since the initial licensing of infliximab, its efficacy and safety have been well-established in diverse patient populations [4–8]. However, high direct costs, constrained healthcare budgets, and stringent reimbursement criteria mean that access to biologic drugs such as infliximab may be limited for some patients for whom this treatment is recommended [9].

A biosimilar is a biologic agent that is concluded to be highly similar to a licensed reference biologic drug [10, 11]. To obtain regulatory approval, a biosimilar undergoes rigorous comparative evaluation with the reference biologic. This biosimilarity exercise includes analytical (structural and functional) characterization and assessment of clinical pharmacokinetics and safety, often conducted in healthy subjects (and preceded, if required, by nonclinical studies). This is followed by a comparative clinical study to confirm that any differences identified earlier in the development program are not clinically meaningful with regard to efficacy, safety, pharmacokinetics, and immunogenicity. By choosing a relevant patient population, confirmatory evidence obtained from this trial forms the basis for extrapolation of data for the biosimilar and its authorization in other indications for which the reference product is approved, without the need to perform additional clinical trials [10, 11].

Originally developed by Pfizer, PF-06438179/GP1111 (PF-SZ-IFX) is an infliximab biosimilar that is approved in the EU [12], Japan [13], the USA [14], and Brazil [15] for all eligible indications of reference infliximab (Remicade^®^) in each region. In preclinical studies, when compared with reference infliximab, PF-SZ-IFX was shown to have an identical primary amino acid sequence and similar biologic activity, including binding to TNF and inhibition of TNF-induced cell apoptosis in vitro [16]; in studies conducted in healthy subjects, PF-SZ-IFX also exhibited similarity to reference infliximab in its pharmacokinetic, safety, and immunogenicity profiles [17].

In view of the relatively truncated development pathway with respect to that for the reference biologic, biosimilars offer potential savings on cost, and their adoption can expand patients’ access to effective biologic therapy, potentially providing considerable health benefits from both a patient and a societal perspective [18–23]. Implementation of postmarketing pharmacovigilance or risk-management plans is frequently a key regulatory requirement of biosimilar manufacturers [24]. While such initiatives greatly expand the knowledge of and experience with their products over time, acquiring data on switching and on the longer-term efficacy and safety of biosimilars in the clinical trial setting (beyond that required to support regulatory approval) is valuable in instilling patient and clinician confidence in their use.

REFLECTIONS B537-02 was a phase III, double-blind, randomized, active-controlled 78-week trial conducted to compare the efficacy, safety, and immunogenicity of reference infliximab sourced from the EU (IFX-EU) and PF-SZ-IFX in patients with moderate-to-severe active rheumatoid arthritis (RA) and an inadequate response to methotrexate. The therapeutic equivalence of IFX-EU and PF-SZ-IFX was confirmed in the initial 30-week treatment period (TP) of the study (TP1), as the 95% confidence intervals (CIs) for the treatment difference in the primary endpoint (American College of Rheumatology [ACR] criteria for ≥ 20% clinical improvement [ACR20] response at week 14) between IFX-EU and PF-SZ-IFX were within prespecified margins [25]. Findings from TP2 (30–54 weeks) showed that similarity between IFX-EU and PF-SZ-IFX was maintained for up to 54 weeks and was not influenced by a single, blinded transition from IFX-EU to PF-SZ-IFX at 30 weeks [26]. Here, we present longer-term efficacy, safety, and immunogenicity results from weeks 54 to 78 (TP3), in which all patients received open-label treatment with PF-SZ-IFX.

## Methods

The methodology of the REFLECTIONS B537-02 study (ClinicalTrials.gov identifier NCT02222493; EudraCT number 2013-004148-49) has been described in detail in previous publications [25, 26] and is briefly summarized here.

### Study Conduct

The study was conducted in accordance with the ethical principles of the Declaration of Helsinki and in compliance with International Conference on Harmonisation Good Clinical Practice guidelines. The independent ethics committee or institutional review board for each study center approved the final study protocol; an independent data monitoring committee was responsible for monitoring safety and study conduct during the blinded portion of the study. All patients provided written informed consent before study entry (no additional informed consent was required because the three treatment periods were part of the same study).

### Patients

Eligibility criteria have been described previously [25]. Briefly, eligible patients were adults (aged ≥ 18 years) who satisfied the 2010 ACR/European League Against Rheumatism (EULAR) classification criteria for RA for ≥ 4 months and ACR classes I–III functional status, based on the 1991 revised criteria [27, 28]. They had moderate-to-severe active RA, with six or more tender and six or more swollen joints and a high-sensitivity C-reactive protein (hs-CRP) level ≥ 10 mg/L despite treatment with oral or parenteral methotrexate at doses of 10–25 mg/week for ≥ 12 weeks. Patients were excluded if they were currently receiving or had previously received treatment with infliximab or a lymphocyte-depleting therapy (e.g., rituximab). Treatment with up to two doses of a nondepleting, non-infliximab biologic was permitted if the biologic had been discontinued ≥ 12 weeks or five half-lives (whichever was longer) before the patient received the first dose of study drug.

### Study Design and Treatments

**Fig. 1:**
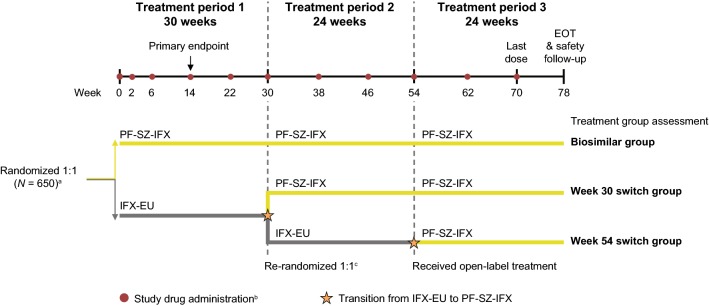
Study design. ^a^Stratified according to geographic region (North America and Western Europe, Japan, Republic of Korea, Latin America, and the rest of the world). ^b^3 mg/kg intravenously. The treatment dose could be increased to 5 mg/kg and the escalated dose maintained in patients with an inadequate response at or after week 14. ^c^In a blinded manner, without stratification. *EOT* end of treatment, *IFX*-*EU* reference infliximab sourced from the EU, *PF*-*SZ*-*IFX* PF-06438179/GP1111, *RA* rheumatoid arthritis

This multinational, randomized, double-blind, active-controlled study comprised three TPs (Fig. 1). At the start of TP1, patients stratified by geographic region were randomized (1:1) to receive blinded treatment with PF-SZ-IFX or IFX-EU at an intravenous dose of 3 mg/kg administered at weeks 0, 2, and 6, and then every 8 weeks; TP1 ended with the completion of week-30 pre-dose assessments. The treatment dose could be increased to 5 mg/kg, and the escalated dose maintained, in patients with an inadequate response at or after week 14. At week 30, the beginning of TP2, patients treated with PF-SZ-IFX in TP1 continued to receive PF-SZ-IFX every 8 weeks; patients treated with IFX-EU in TP1 were re-randomized (1:1), without stratification and in a blinded fashion, to either continue receiving IFX-EU or switch to PF-SZ-IFX; TP2 ended with the completion of week-54 pre-dose assessments. At week 54, the beginning of TP3, all patients received open-label treatment with PF-SZ-IFX, which was continued until the end of the study; the last study dose in TP3 was administered at week 70, and the last study visit was at week 78. Patients continued to receive stable doses of methotrexate and folic/folinic acid throughout the study.

### Assessments

As reported previously, the primary efficacy endpoint was the proportion of patients achieving ACR20 response at week 14 [25]. Therapeutic equivalence was demonstrated with the two-sided 95% CI for the treatment difference in ACR20 response rates falling within the prespecified symmetric equivalence margin of ± 13.5%.

In TP3, secondary efficacy endpoints assessed at weeks 62, 70, and 78 included the proportions of patients who achieved ACR criteria for ≥ 20%/≥ 50%/≥ 70% improvement (ACR20/ACR50/ACR70 response); EULAR response; remission based on Disease Activity Score 28 joint count CRP (DAS28-CRP) criterion (i.e., DAS28-CRP < 2.6), and on ACR/EULAR criteria (i.e., tender joint count ([TJC] and swollen joint count [SJC] ≤ 1, hs-CRP level ≤ 1 mg/dL, and patient global assessment score ≤ 1; or Simplified Disease Activity Index ≤ 3.3). Changes from study baseline in DAS28-CRP, TJC, and SJC, hs-CRP, and Health Assessment Questionnaire—Disability Index (HAQ-DI) were also assessed at these time points.

Safety and tolerability were evaluated throughout TP3 based on the reporting of adverse events (AEs), including treatment-emergent adverse events (TEAEs) and serious AEs (SAEs). AEs were coded according to the Medical Dictionary for Regulatory Activities (version 20.0) classification system; AE severity was graded according to the National Cancer Institute Common Terminology Criteria for Adverse Events (version 4.03).

Immunogenicity was assessed based on the number and percentage of patients in TP3 who had one or more post-dose samples that tested positive for antidrug antibodies (ADAs) or neutralizing antibodies (NAbs) in ADA-positive samples. Serum samples were analyzed for ADAs with a validated electrochemiluminescence assay using a tiered approach (i.e., screening, confirmation, and titer/quantitation). Additional details regarding immunogenicity testing in this study were reported previously [25]. Serum trough concentrations of PF-SZ-IFX in TP3 were analyzed in all patients and by ADA-positive and ADA-negative subgroups.

### Statistical Methods

Treatment efficacy in TP3 was analyzed in the intent-to-treat (ITT) population, which included all patients enrolled and treated with one or more doses of study drug in TP3. Efficacy data were summarized using descriptive statistics for the ITT population. Safety and immunogenicity data were summarized descriptively for the safety population, which comprised all randomized patients who received one or more doses of study drug in TP3. Analyses were based on observed data collected in TP3; no imputation was applied to missing data during TP3.

Data were analyzed for all patients and were evaluated in three groups in TP3 corresponding to the treatment sequence in TP1/TP2/TP3: biosimilar group (PF-SZ-IFX/PF-SZ-IFX/PF-SZ-IFX), week 30 switch group (IFX-EU/PF-SZ-IFX/PF-SZ-IFX), and week 54 switch group (IFX-EU/IFX-EU/PF-SZ-IFX) (Fig. 1).

Summary statistics for serum trough concentrations of PF-SZ-IFX were calculated by setting concentration values below the lower limit of quantification (LLOQ) to 0 (LLOQ = 100 ng/mL).

## Results

### Patient Disposition and Baseline Characteristics

As previously reported, 650 patients were initially randomized to PF-SZ-IFX (*n* = 324) or IFX-EU (*n* = 326) in TP1, and 566 patients who completed the initial period entered TP2 at week 30 [25, 26]. A total of 505 patients participated in TP3, comprising 253 patients in the biosimilar group, 126 patients in the week 30 switch group, and 126 patients in the week 54 switch group (Table 1). Of these, 470 (93.1%) patients completed TP3, with comparable completion rates observed in the three groups (93.7% in the biosimilar group, 92.9% in the week 30 switch group, and 92.1% in the week 54 switch group). In this TP, 35.6%, 33.3%, and 32.5% of the patients in the biosimilar group, week 30 switch group, and week 54 switch group, respectively, received at least one escalated dose of the study drug (5 mg/kg).

**Table 1:** Patient disposition through TP3 (ITT population)

Patient disposition	Biosimilar group (*n* = 253)	Week 30 switch group (*n* = 126)	Week 54 switch group (*n* = 126)	Total (*N* = 505)
Treated during TP3	253 (100.0)	126 (100.0)	126 (100.0)	505 (100.0)
Discontinued from study	16 (6.3)	9 (7.1)	10 (7.9)	35 (6.9)
Completed study	237 (93.7)	117 (92.9)	116 (92.1)	470 (93.1)

Data are presented as *n* (%) unless otherwise indicated

*ITT* intent-to-treat, *N* number of patients in the TP3 ITT population, *n* number of patients in each category, *TP3* treatment period 3

Baseline demographics and RA characteristics were comparable between the three treatment groups in TP3 (Table 2). Most patients were female (79.2%) and White (78.6%), and the average age was 52.4 years.

**Table 2:** Demographics and clinical characteristics of patients participating in TP3 at week 54 (ITT population)

Characteristics	Biosimilar group (*n* = 253)	Week 30 switch group (*n* = 126)	Week 54 switch group (*n* = 126)	Total (*N* = 505)
*Demographic characteristics*				
Female	198 (78.3)	104 (82.5)	98 (77.8)	400 (79.2)
Age, years	52.4 ± 12.8	51.3 ± 12.6	53.5 ± 12.4	52.4 ± 12.7
Weight, kg	73.3 ± 19.7	74.5 ± 18.0	73.1 ± 21.0	73.6 ± 19.6
BMI, kg/m^2^	27.1 ± 6.3	27.9 ± 7.1	27.1 ± 6.7	27.3 ± 6.6
Race				
White	207 (81.8)	97 (77.0)	93 (73.8)	397 (78.6)
Asian	36 (14.2)	16 (12.7)	18 (14.3)	70 (13.9)
Ethnicity				
Not Hispanic/Latino	236 (93.3)	111 (88.1)	116 (92.1)	463 (91.7)
Geographic region				
North America and Western Europe	33 (13.0)	17 (13.5)	15 (11.9)	65 (12.9)
Japan	19 (7.5)	9 (7.1)	7 (5.6)	35 (6.9)
Republic of Korea	2 (0.8)	0 (0.0)	2 (1.6)	4 (0.8)
Latin America	13 (5.1)	9 (7.1)	9 (7.1)	31 (6.1)
Rest of the world	186 (73.5)	91 (72.2)	93 (73.8)	370 (73.3)
*Disease characteristics*				
RA duration, years	7.4 ± 8.8	6.1 ± 6.3	6.9 ± 7.3	7.0 ± 7.9
Swollen joint count	16.3 ± 9.7	16.4 ± 9.2	15.3 ± 7.7	16.1 ± 9.1
Tender joint count	24.4 ± 13.6	25.4 ± 13.1	24.8 ± 11.9	24.8 ± 13.1
hs-CRP, mg/L	25.7 ± 23.7	27.2 ± 35.2	23.1 ± 21.9	25.4 ± 26.6
*Concomitant medication*				
MTX dose, mg/week	14.0 ± 4.2	14.5 ± 4.1	14.2 ± 4.9	14.2 ± 4.4

Data are presented as mean ± standard deviation or *N* (%) unless otherwise indicated

*BMI* body mass index, *hs*-*CRP* high-sensitivity C-reactive protein, *ITT* intent-to-treat, *MTX* methotrexate, *N* number of patients in the TP3 ITT population, *n* number of patients in each category, *RA* rheumatoid arthritis, *TP3* treatment period 3

### Efficacy

**Fig. 2:**
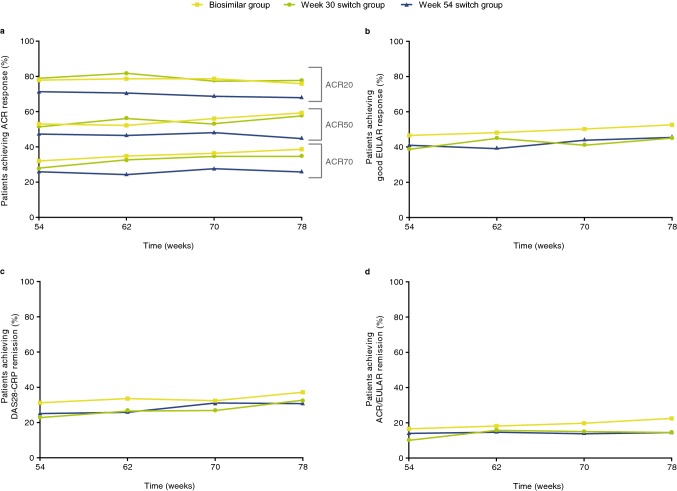
Proportions of patients achieving **a** ACR20/50/70 responses, **b** good EULAR response, **c** DAS28-CRP remission, and **d** ACR/EULAR remission during TP3. ITT population in TP3. *ACR20/50/70* 20%/50%/70% improvement in American College of Rheumatology response from study baseline (week 0), *DAS28-CRP* Disease Activity Score in 28 joints based on high-sensitivity C-reactive protein, *EULAR* European League Against Rheumatism, *ITT* intent-to-treat, *TP3* treatment period 3

In TP1, the primary endpoint of the study of ACR20 response at week 14 was met and therapeutic equivalence between PF-SZ-IFX and IFX-EU demonstrated, since the 95% CI for the between-treatment-group difference in ACR20 response rates at week 14 was contained within the prespecified symmetric equivalence margins [25]. ACR20 response rates for patients in the biosimilar group, week 30 switch group, and week 54 switch group were 77.9%, 78.6%, and 71.4% before the first infusion of study drug in TP3, and 75.9%, 77.8%, and 68.3%, respectively, at week 78. At week 54, before the first infusion of PF-SZ-IFX in TP3, 76.4%, 51.3%, and 29.5% of all patients who were evaluated in TP3 achieved ACR20, ACR50, and ACR70 responses, respectively; 74.5%, 55.5%, and 34.7% of all patients achieved these responses at week 78, respectively. The proportions of patients with ACR20/50/70 responses were overall comparable among the three treatment groups at all study visits between weeks 54 and 78 (Fig. 2a). Rates of good EULAR response were 43.4% and 49.1% at weeks 54 and 78, respectively, in all patients; these rates were also comparable among the three treatment groups in TP3 (Fig. 2b).

Remission based on DAS28-CRP and ACR/EULAR criteria was achieved in 27.7% and 14.5% of all patients, respectively, at week 54 and in 34.5% and 18.6% of patients at week 78. As with clinical response rates, remission rates were sustained and comparable among the three treatment groups during TP3 (Fig. 2c, d).

**Fig. 3:**
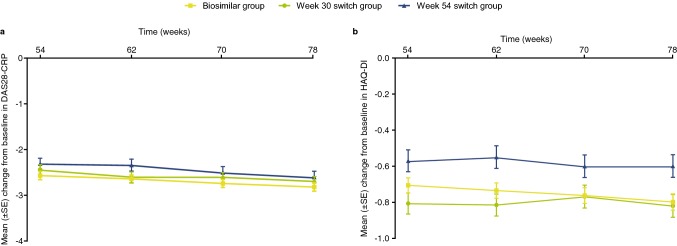
Mean changes from study baseline (week 0) in **a** DAS28-CRP and **b** HAQ-DI during TP3. ITT population in TP3. *DAS28*-*CRP* Disease Activity Score in 28 joints based on high-sensitivity C-reactive protein, *HAQ*-*DI* Health Assessment Questionnaire—Disability Index, *ITT* intent-to-treat, *SE* standard error

At week 54, the mean DAS28-CRP in all patients was 3.5, reflecting a mean change from study baseline (week 0) of − 2.5; at week 78, the mean DAS28-CRP in all patients was 3.2, reflecting a mean change from baseline of − 2.7. Throughout TP3, mean changes from baseline in DAS28-CRP were overall comparable among the three treatment groups (Fig. 3a). The mean HAQ-DI score at week 54 in all patients was 0.9, for a mean change from study baseline of − 0.7; the mean HAQ-DI score at week 78 was 0.8, for a mean change from baseline of − 0.8. As with response and remission rates and changes in DAS28-CRP, changes from baseline in HAQ-DI scores were comparable among the three treatment groups in TP3 (Fig. 3b).

### Safety

Across all three treatment groups, the median duration of treatment from the first infusion in TP1 to the last infusion in TP3 was 70.1 weeks. Drug exposure was similar among the three treatment groups in TP3. The mean (± standard deviation) total dose administered was 787.7 ± 321.5 mg, 790.4 ± 319.1 mg, and 761.8 ± 368.6 mg for patients in the biosimilar group, week 30 switch group, and week 54 switch group, respectively. No patient in any treatment group required a dose reduction in TP3 because of an AE.

In TP3, a total of 148 (29.3%) patients reported TEAEs and 12 (2.4%) reported SAEs (Table 3). Treatment-related SAEs were reported by four (0.8%) patients: in the week 30 switch group, one patient experienced cellulitis, one reported chronic sinusitis and encephalitis, and one patient experienced tuberculosis; one patient in the week 54 switch group experienced endometriosis. Six (1.2%) patients were discontinued from the study as a result of AEs. Among all patients in the safety population from TP3, 67 (13.3%) reported an infectious TEAE; of these, four (0.8%) and five (1.0%) reported a serious infectious TEAE and a grade 3 infectious TEAE, respectively. The most common TEAEs in the three treatment groups were viral upper respiratory tract infections, upper respiratory tract infections, infusion-related reactions, exacerbation of RA, and oropharyngeal pain. The incidences of TEAEs and SAEs during TP3 were comparable between treatment groups.

**Table 3:** All-cause TEAEs in patients participating in treatment period 3

	Biosimilar group (*n* = 253)	Week 30 switch group (*n* = 126)	Week 54 switch group (*n* = 126)	Total (*N* = 505)
Number of AEs	117	64	69	250
Patients with events				
AEs	73 (28.9)	37 (29.4)	38 (30.2)	148 (29.3)
SAEs	3 (1.2)	6 (4.8)	3 (2.4)	12 (2.4)
Grade 3 AEs	4 (1.6)	7 (5.6)	3 (2.4)	14 (2.8)
Grade 4 AEs	1 (0.4)	0	0	1 (0.2)
Grade 5 AEs	0	0	0	0
Patients who were discontinued because of AEs				
From treatment, temporarily	1 (0.4)	6 (4.8)	4 (3.2)	11 (2.2)
From treatment, permanently	5 (2.0)	2 (1.6)	2 (1.6)	9 (1.8)
From study	3 (1.2)	1 (0.8)	2 (1.6)	6 (1.2)
AEs occurring in ≥ 2% of patients in any treatment group				
Upper respiratory tract infection	5 (2.0)	4 (3.2)	4 (3.2)	13 (2.6)
Viral upper respiratory tract infection	11 (4.3)	4 (3.2)	5 (4.0)	20 (4.0)
Infusion-related reaction	3 (1.2)	3 (2.4)	4 (3.2)	10 (2.0)
Rheumatoid arthritis	2 (0.8)	2 (1.6)	3 (2.4)	7 (1.4)
Oropharyngeal pain	0	3 (2.4)	0	3 (0.6)
Patients with AEs of special interest				
Infusion-related reaction	3 (1.2)	3 (2.4)	4 (3.2)	10 (2.0)
Hypersensitivity	9 (3.6)	6 (4.8)	6 (4.8)	21 (4.2)
Infections	32 (12.6)	19 (15.1)	16 (12.7)	67 (13.3)
Tuberculosis	0	1 (0.8)	0	1 (0.2)
Pneumonia^a^	1 (0.4)	1 (0.8)	1 (0.8)	3 (0.6)
Neoplasms	2 (0.8)	1 (0.8)	0	3 (0.6)
Malignancies^b^	1 (0.4)	0	0	1 (0.2)

Data are presented as *n* (%) unless otherwise indicated

*AE* adverse event, *N* number of patients in final TP safety population, *n* number of patients in each category, *SAE* serious adverse event, *TEAE* treatment-emergent adverse event, *TP* treatment period

^a^Includes one patient with atypical pneumonia (week 30 switch group)

^b^One patient developed transitional bladder cancer, which was not considered to be related to the study drug

### Immunogenicity

**Fig. 4:**
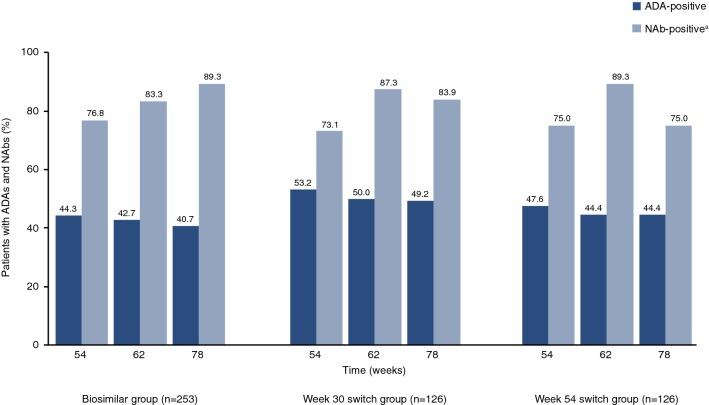
The proportions of patients who tested positive for ADAs and, of those, the proportions who tested positive for NAbs, by study visit in TP3. ^a^NAb-positive incidences are expressed as percent of ADA-positive patients. *ADA* antidrug antibody, *NAb* neutralizing antidrug antibody, *TP3* treatment period 3

Overall, ADAs were detected in 119 (47.0%), 72 (57.1%), and 66 (52.4%) patients in the biosimilar group, week 30 switch group, and week 54 switch group, respectively, during TP3, regardless of their ADA status in TP1 and TP2. Among patients who tested positive for ADAs, 105 (88.2%), 60 (83.3%), and 58 (87.9%) also tested positive for NAbs in the three treatment groups, respectively. The proportions of patients who were ADA positive and NAb positive, regardless of their previous ADA status, at week 54 and week 78 (post-dose) were comparable among the three treatment groups (Fig. 4).

Of 505 patients who entered TP3, 213 (42.2%) did not have a prior post-dose ADA-positive test. Of these ADA-negative patients, 14 (6.6%) had their first post-dose ADA-positive test during TP3, comprising 6.0%, 6.4%, and 8.0% of patients in the biosimilar group, week 30 switch group, and week 54 switch group, respectively.

Patients who were ADA positive had lower mean serum trough concentrations of PF-SZ-IFX than patients who were ADA negative (Fig. S1 in the electronic supplementary material). However, within the ADA-positive and ADA-negative subgroups, mean concentrations were generally comparable across treatment groups during TP3.

The majority of patients who developed ADAs did not report TEAEs of hypersensitivity or infusion-related reactions during TP3. Of the 306 patients who tested positive for ADAs during all treatment periods (144, 82, and 80 patients in the biosimilar group, week 30 switch group, and week 54 switch group, respectively), ten (3.3%) experienced TEAEs of hypersensitivity in TP3 (three [2.1%], four [4.9%], and three [3.8%], respectively) and nine (2.9%) experienced TEAEs of infusion-related reactions (three [2.1%], three [3.7%], and three [3.8%], respectively). None of the TEAEs of hypersensitivity events or infusion-related reactions were considered serious or above grade 2 in severity. One patient from each treatment group (0.4%, 0.8%, and 0.8%, respectively) was withdrawn from the study as a result of TEAEs of hypersensitivity. Two (1.4%), one (1.2%), and one (1.3%) patients in these groups, respectively, were withdrawn because of TEAEs of infusion-related reactions on or after ADA detection.

## Discussion

The earlier findings from the REFLECTIONS B537-02 study in relation to the primary endpoint confirmed the therapeutic equivalence of PF-SZ-IFX and reference infliximab [25] and contributed to the “totality of the evidence” in support of the regulatory approval of PF-SZ-IFX in the treatment of patients with RA, as well as all other eligible indications for which reference infliximab is authorized [29]. The data reported here, obtained during TP3 from the same study, provide additional valuable clinical evidence concerning switching patients with RA from treatment with IFX-EU to PF-SZ-IFX as well as on the effects of longer-term treatment with PF-SZ-IFX. In this respect, the efficacy of PF-SZ-IFX, as judged by ACR responses (Fig. 2a), was comparable across groups during TP3, with no clinically meaningful differences between patients maintained on PF-SZ-IFX throughout the 78 weeks of the study (biosimilar group) and those who switched from IFX-EU (week 30 and week 54 switch groups). Comparability across treatment groups was also evident from assessment of other secondary clinical outcome measures, such as EULAR response (Fig. 2b) and DAS28-CRP (Fig. 2c) and ACR/EULAR remission criteria (Fig. 2d).

For patients who switched from IFX-EU, there was no clinically meaningful difference in ACR20 response from the time of the last treatment with IFX-EU to the end of the study (week 30 switch group: 75.5% [week 30] [26] and 77.8% [week 78]; week 54 switch group: 71.4% [week 54] and 68.3% [week 78]). For patients receiving PF-SZ-IFX before entry to TP3, ACR20 responses at the end of double-blind treatment (TP2) were sustained during open-label treatment in TP3 (biosimilar group: 77.9% [week 54] and 75.9% [week 78]; week 30 switch group: 78.6% [week 54] and 77.8% [week 78]). This profile, with respect to ACR20 responses, was also reflected in other secondary efficacy outcome measures.

Overall, the safety profile was comparable between treatment groups and was consistent with the known long-term safety profile for infliximab in patients with RA [30]. There were no noticeable differences in the proportions of patients experiencing AEs between patients maintained on PF-SZ-IFX throughout the 78 weeks of the study (biosimilar group; 28.9%) and those who switched from IFX-EU (week 30 [29.4%] and week 54 switch groups [30.2%]). The incidence of SAEs, and AEs leading to study discontinuation, was also comparable among the three treatment groups during TP3. Moreover, there was no clinically meaningful difference between groups in the frequency of AEs of special interest reported, including infusion-related reactions, hypersensitivity, and infections.

Immunogenicity assessment during TP3 showed the incidence of both ADA and NAb development was comparable between groups, with no clinically meaningful differences in the proportions of patients who tested positive for ADAs and who showed NAb positivity. Overall, the safety and immunogenicity findings suggested cumulative exposure to PF-SZ-IFX for up to 78 weeks (biosimilar group) or following switching from IFX-EU, and follow-up for up to 48 weeks (week 30 and week 54 switch groups) did not increase the occurrence of AEs or immunogenicity or adversely affect the safety profile.

Limitations of the study include the absence of patients maintained on IFX-EU throughout as a control group. No formal hypothesis testing was conducted for any of the secondary endpoints; therefore, results were interpreted based on descriptive statistics.

## Conclusions

Results from TP3 of the REFLECTIONS B537-02 study showed patients with moderate-to-severe active RA receiving PF-SZ-IFX experienced no clinically meaningful differences in efficacy, safety, or immunogenicity, regardless of whether they were maintained on PF-SZ-IFX throughout 78 weeks of treatment or following single treatment transitions from IFX-EU to PF-SZ-IFX at week 30 or at week 54. PF-SZ-IFX was well-tolerated for up to 78 weeks of treatment and displayed a safety profile consistent with that of infliximab. These findings provide long-term clinical data to complement the existing evidence of similarity between reference infliximab and PF-SZ-IFX, including structure, biological function, pharmacokinetics, and therapeutic equivalence, and add to the “totality of the evidence” supporting biosimilarity of PF-SZ-IFX to reference infliximab and its use in the other eligible indications for which reference infliximab is authorized.

## Electronic supplementary material

Below is the link to the electronic supplementary material.

Supplementary material 1 (PDF 348 kb)

## Data Availability

Upon request, and subject to certain criteria, conditions, and exceptions (see https://www.pfizer.com/science/clinical-trials/trial-data-and-results for more information), Pfizer will provide access to individual de-identified participant data from Pfizer-sponsored global interventional clinical studies conducted for medicines, vaccines, and medical devices (1) for indications that have been approved in the USA and/or EU or (2) in programs that have been terminated (i.e., development for all indications has been discontinued). Pfizer will also consider requests for the protocol, data dictionary, and statistical analysis plan. Data may be requested from Pfizer trials 24 months after study completion. The de-identified participant data will be made available to researchers whose proposals meet the research criteria and other conditions, and for which an exception does not apply, via a secure portal. To gain access, data requestors must enter into a data access agreement with Pfizer.
