# Supplementary material for: Long-term Efficacy, Safety, and Immunogenicity of the Infliximab (IFX) Biosimilar, PF-06438179/GP1111, in Patients with Rheumatoid Arthritis After Switching from Reference IFX or Continuing Biosimilar Therapy: Week 54–78 Data From a Randomized, Double-Blind, Phase III Trial
Source: BioDrugs. 2020 Jan 14;34(2):197–207. doi: 10.1007/s40259-019-00403-z (PMC7113200; doi:10.1007/s40259-019-00403-z)

Electronic Supplementary Material

Article title: **Long-term Efficacy, Safety, and Immunogenicity of the Infliximab (IFX) Biosimilar, PF-06438179/GP1111, in Patients with Rheumatoid Arthritis After Switching from Reference IFX or Continuing Biosimilar Therapy: Week 54–78 Data from a Randomized, Double-Blind, Phase III Trial**

Journal: **BioDrugs**

Authors: Stanley B. Cohen, Sebastiao C. Radominski, Hideto Kameda, Alan J. Kivitz, Michael Tee, Carol Cronenberger, Min Zhang, Sarah Hackley, Muhammad I. Rehman, Oliver von Richter, Rieke Alten

Corresponding author: Stanley B. Cohen, Metroplex Clinical Research Center, Dallas, TX 75231, USA; Email arthdoc@aol.com

Fig. S1 Serum trough concentrations of PF-SZ-IFX by treatment group over time in treatment period 3 in **a)** all patients, **b)** patients who were ADA-positive, and **c)** patients who were ADA-negative

PK population in treatment period 3
ADA anti-drug antibody, *PF-SZ-IFX* PF-06438179/GP1111, *PK* pharmacokinetic,
*Box plots provide medians and 25%/75% quartiles; whiskers indicate the last point that is within 1.5-times of the interquartile range

Fig. S1

a All subjects

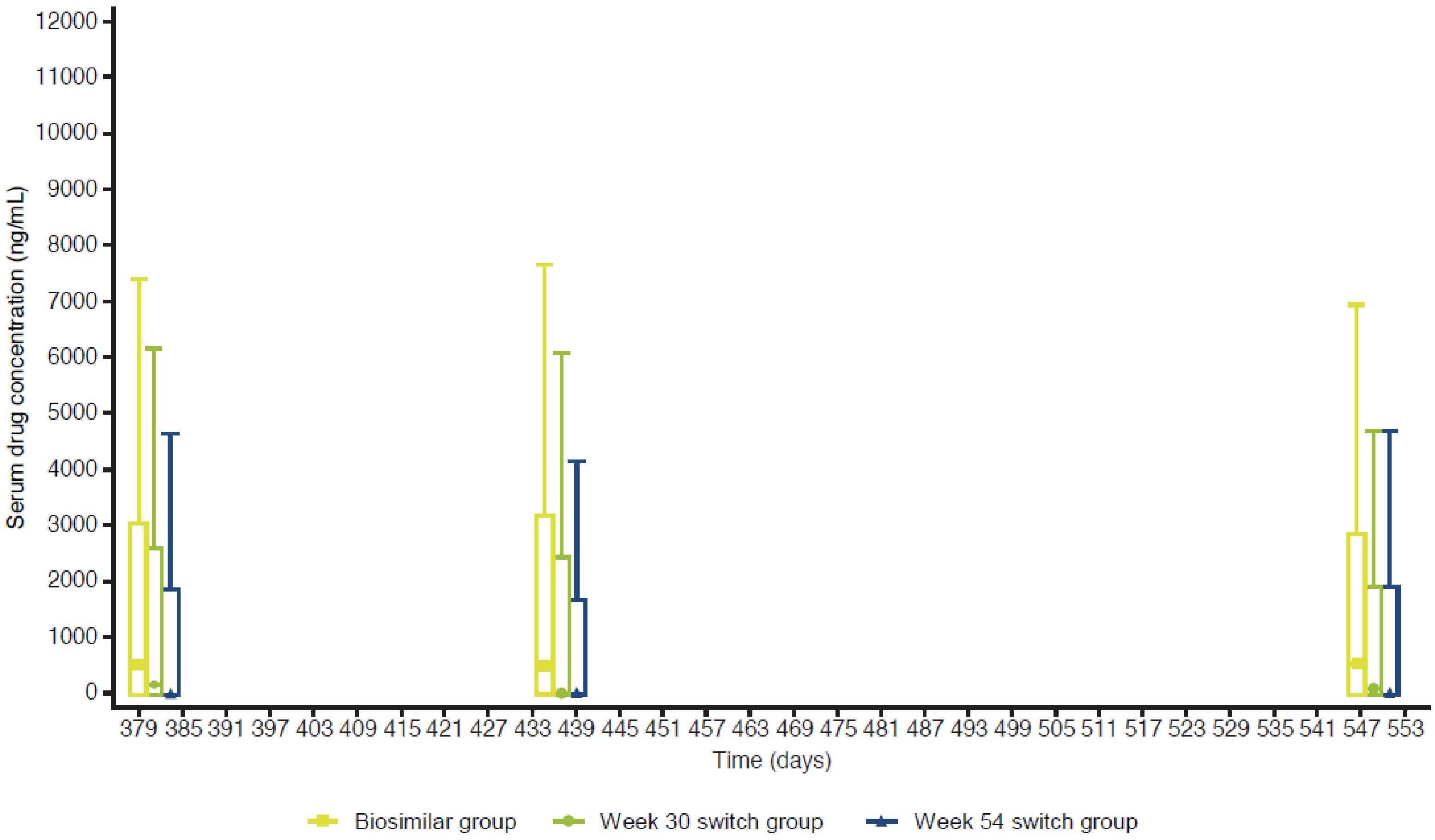

b ADA-positive subjects

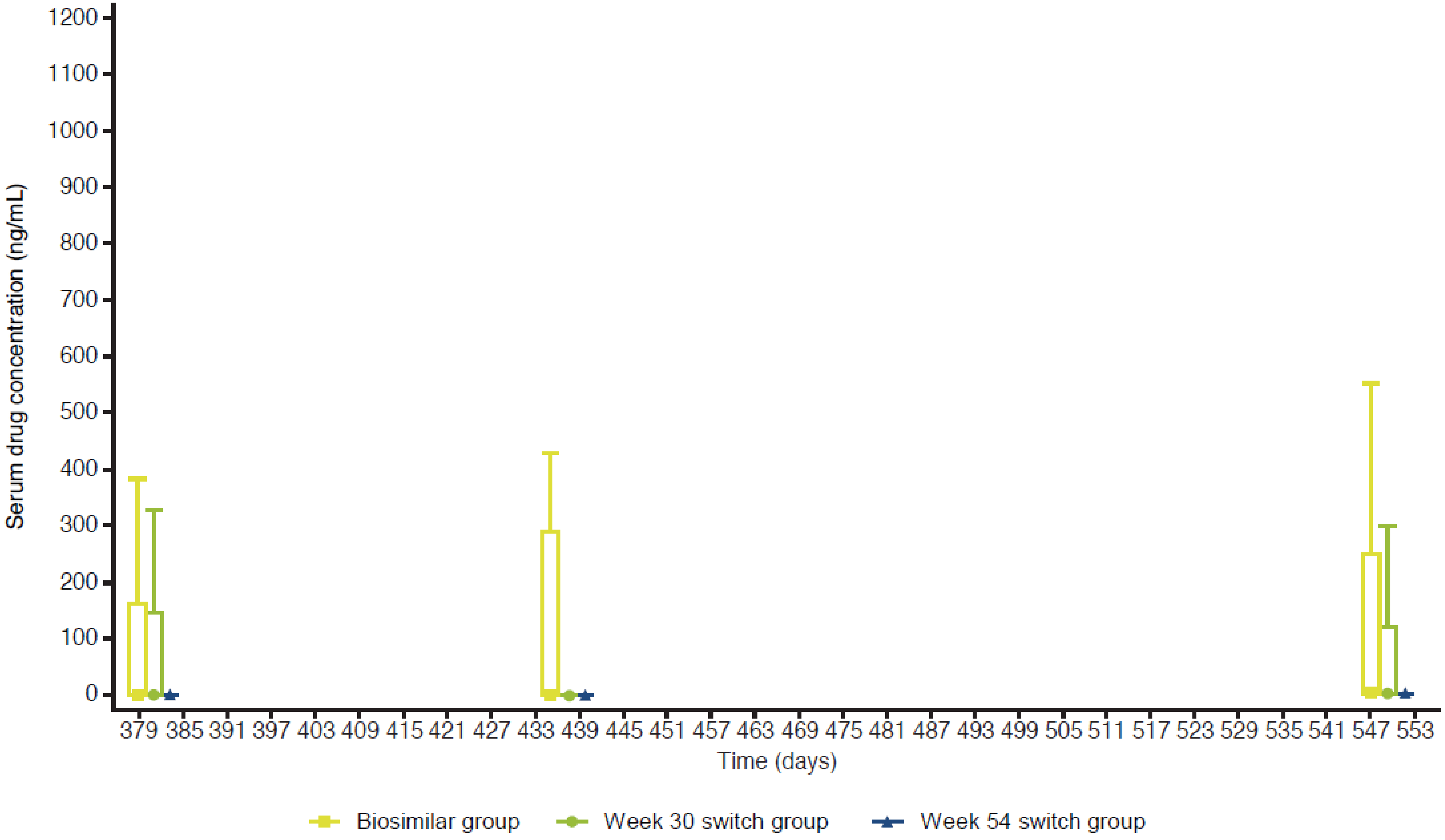

c ADA-negative subjects

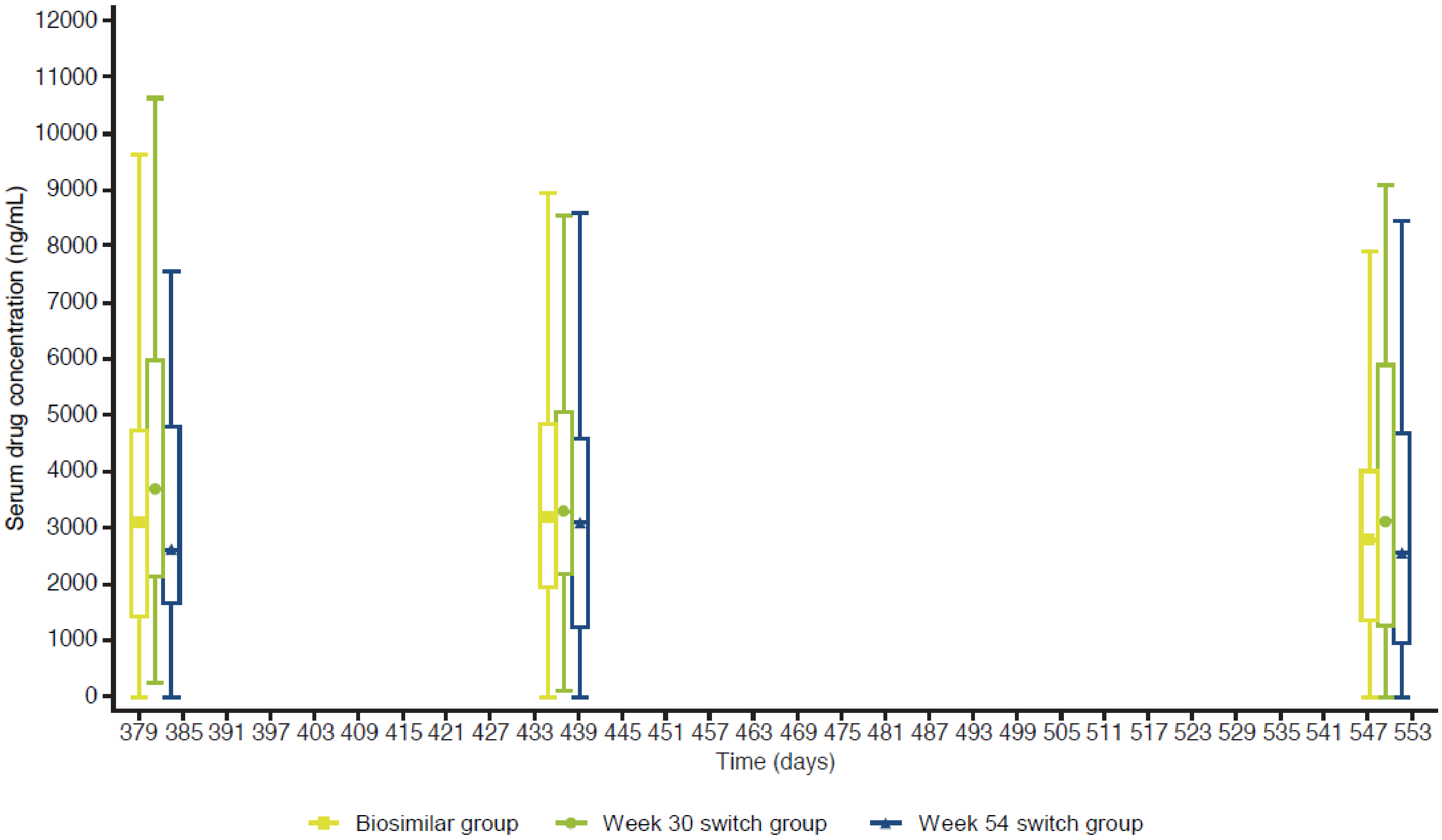

Supplement: Supplementary file 1 — Supplementary material 1 (PDF 348 kb) [file 40259_2019_403_MOESM1_ESM.pdf]
